# A dynamic model of the body gas stores for carbon dioxide, oxygen, and inert gases that incorporates circulatory transport delays to and from the lung

**DOI:** 10.1152/japplphysiol.00764.2020

**Published:** 2021-01-21

**Authors:** Snapper R. M. Magor-Elliott, Christopher J. Fullerton, Graham Richmond, Grant A. D. Ritchie, Peter A. Robbins

**Affiliations:** ^1^Department of Physiology, Anatomy and Genetics, University of Oxford, United Kingdom;; ^2^Department of Chemistry, Physical and Theoretical Chemistry Laboratory, University of Oxford, United Kingdom

**Keywords:** mixed venous composition, mixed venous saturation, pulmonary arterial blood gases

## Abstract

Many models of the body’s gas stores have been generated for specific purposes. Here, we seek to produce a more general purpose model that: *1*) is relevant for both respiratory (CO_2_ and O_2_) and inert gases; *2*) is based firmly on anatomy and not arbitrary compartments; *3*) can be scaled to individuals; and *4*) incorporates arterial and venous circulatory delays as well as tissue volumes so that it can reflect rapid transients with greater precision. First, a “standard man” of 11 compartments was produced, based on data compiled by the International Radiation Protection Commission. Each compartment was supplied via its own parallel circulation, the arterial and venous volumes of which were based on reported tissue blood volumes together with data from a detailed anatomical model for the large arteries and veins. A previously published model was used for the blood gas chemistry of CO_2_ and O_2_. It was not permissible ethically to insert pulmonary artery catheters into healthy volunteers for model validation. Therefore, validation was undertaken by comparing model predictions with previously published data and by comparing model predictions with experimental data for transients in gas exchange at the mouth following changes in alveolar gas composition. Overall, model transients were fastest for O_2_, intermediate for CO_2_, and slowest for N_2_. There was good agreement between model estimates and experimentally measured data. Potential applications of the model include estimation of closed-loop gain for the ventilatory chemoreflexes and improving the precision associated with multibreath washout testing and respiratory measurement of cardiac output.

**NEW & NOTEWORTHY** A model for the body gas stores has been generated that is applicable to both respiratory gases (CO_2_ and O_2_) and inert gases. It is based on anatomical details for organ volumes and blood contents together with anatomical details of the large arteries. It can be scaled to the body size and composition of different individuals. The model enables mixed venous gas compositions to be predicted from the systemic arterial compositions.

## INTRODUCTION

The gas stores of the lung exchange with the atmosphere via pulmonary ventilation and with the rest of the body via pulmonary blood flow. In terms of measurement, the ventilatory exchange is far more accessible than the exchange occurring via the blood. For this reason, both experimental and modeling studies of the lung often make simplifying assumptions in relationship to gas exchange with the blood, for example, that the composition of the blood flowing into the lungs is constant and that there is no recirculation. A more complete approach to the problem would be to include a model of the circulation and systemic body gas stores, including the metabolic consumption of oxygen and production of carbon dioxide. With such an approach, the gas concentrations in the blood flowing into the lung will be generated automatically from past blood gas concentrations leaving the lungs. Our primary objective is to create a physiological model of this process, the circulation and body gas stores (CBGS) model.

There are already a considerable number of existing models of the body gas stores in the literature. Some are concerned with carbon dioxide (1, 2) and others with inert gases (3), including anesthetics (4, 5). However, most do not provide a single integrated model for all gases. Furthermore, many tend to be built from hypothetical compartments whose parameters are set to values that allow a fit to measured data with little or no anatomical basis. In most cases, these models are only concerned with time scales sufficiently long that circulatory delays may be ignored (1–3, 6, 7), although there are some exceptions relating to the anesthetic gases (4, 5). Our aim is to generate a model that has an anatomical/physiological basis to its compartments, that models both circulatory delays and gas stores, and that is applicable to both the respiratory gases and to other gases for which tissue gas solubilities can be obtained. Finally, individual humans differ substantially in size and as a consequence in their physiology and gas storage capacity. Thus a further objective is to develop a model that can be scaled to the size and body composition of an individual. Potential applications of the model are considered in the discussion.

## METHODS

**Figure 1.**
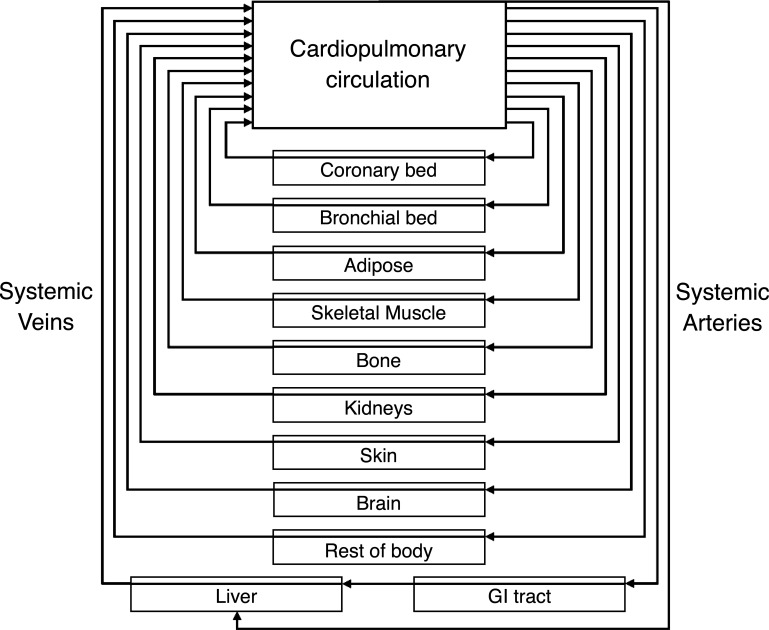
Schematic of the model of the circulatory and body gas stores. Vessels are arranged in parallel flow streams of differing lengths and perfusions. The connected tissues have differing volumes and partial pressure to concentration storage relationships.

The CBGS model consists of a set of independent body compartments, with each connected to the lung by independent parallel circulatory paths (Fig. 1). The arrangement is identical for all tissues apart from the liver and gastrointestinal (GI) tract, which mimic the anatomy of the portal system. In this section we describe the model’s construction, its initialization and execution, and the process used for model validation.

### Construction of a Model for a Standard Man

The compartments of our model for a standard man (male, 1.8 m tall, 70 kg mass, and 25 years old) were based on data from the International Commission for Radiological Protection (ICRP) (8, 9). Relative organ weights from Ref. 8 are as summarized in the review of Brown et al. (4). In our model, we used a single compartment for the GI tract and spleen that feeds into the hepatic portal vein, and condensed the other smaller organs (adrenals, pancreas, and thyroid) into a single, rest of body, compartment. This resulted in a model with a total of 11 compartments, the details of which are given in Table 1. In order that gas concentrations could be expressed per unit volume, the volumes for each compartment were calculated by dividing the mass of each compartment by the relevant tissue density (4, 8).

**Table 1.** Circulation and body gas store parameter values for a standard man

Tissue	Mass /kg	Density /$\mathrm{kg}\cdot L^{-1}$	*Qf*	$V_{c}$ /*L*	$V_{a}$ /*L*	$V_{v}$ /*L*	$t_{D}$ /*s*
Adipose	14.99	0.916	0.050	0.011	0.070	0.266	80.1
Bone	10.01	1.469	0.050	0.011	0.090	0.351	104.2
Brain	1.40	1.036	0.120	0.027	0.039	0.127	18.5
GI tract	1.34	1.046	0.180	0.040	0.127	0.328	31.7
Heart	0.34	1.030	0.040	0.009	0.010	0.039	16.8
Kidneys	0.31	1.050	0.190	0.042	0.056	0.186	17.3
Liver	1.80	1.051	0.065	0.054	0.105	0.573	130.0
Lungs	0.53	1.051	0.025	0.006	0.022	0.089	53.8
Muscle	28.00	1.041	0.170	0.038	0.207	0.780	69.5
Skin	2.60	1.183	0.050	0.011	0.050	0.182	56.0
Blood	5.53	1.060	NaN	NaN	NaN	NaN	NaN
Rest of body	3.16	1.050	0.060	0.013	0.052	0.189	49.0
Citation	(4, 8)	(4, 10, 11)	(4, 12, 13)	(4, 11, 12)	(14–16)	(17, 18)	

*Qf*, fraction of total perfusion; $V_{c}$, capillary volume; $V_{a}$, arterial volume; $V_{v}$, venous volume; $t_{D}$, total transit time through systemic arteries, capillaries, and veins for each compartment.

The vein for the gastrointestinal (GI) tract represents the portal vein supplying the liver.

The values for the liver are for the hepatic artery and vein.

The perfusion and vessel values for the lungs are for the bronchial circulation only.

Reference values for the perfusion and blood volume of each tissue are as recommended by the ICRP (9) (see section 7.7.2, p142). These were derived principally from Refs. 12, 19, and 20. The total systemic capillary volume was assumed to be 5% of the total blood volume (21, 22), and this was distributed between the compartments based on their relative perfusion. The capillary volume for each organ was subtracted from the total blood volume for that organ. This remaining volume was distributed between the arteries and veins for that compartment in a ratio of 1:3. The volumes for each compartment are summarized in Table 1. The effect of body position on these factors was not considered in the model and therefore its influence is unknown.

**Figure 2.**
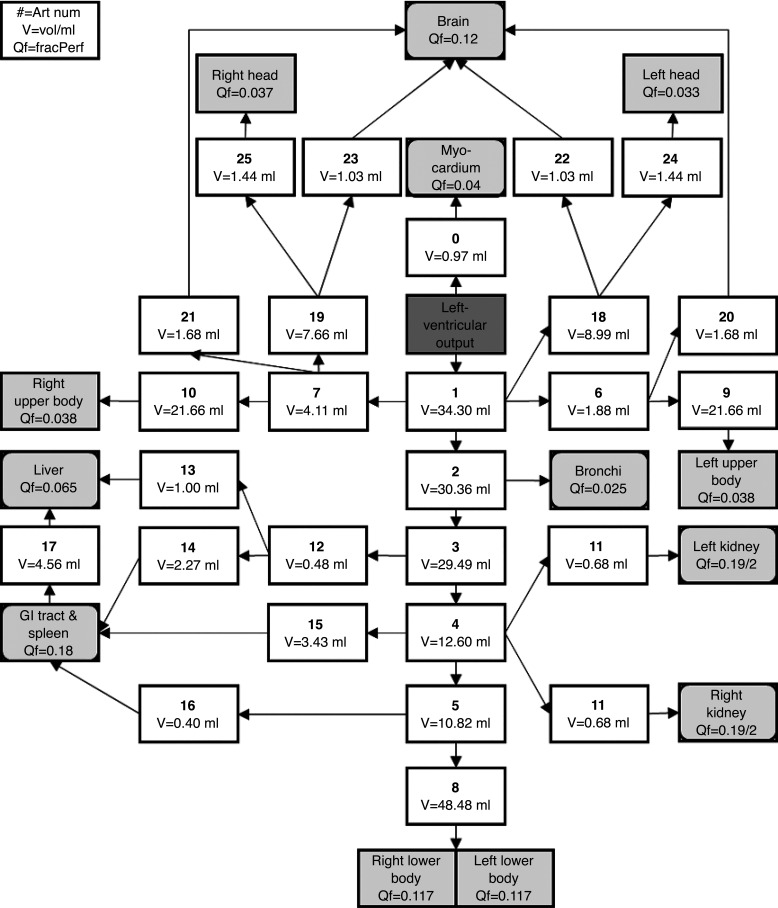
Schematic of the arterial tree used to calculate total vessel volumes for each compartment. Art num, artery number corresponding to the key used in Table A1; V, arterial segment volume/ml; Qf, fraction of the total perfusion supplied to the compartment. White boxes, vessels; light gray rounded boxes, discrete tissues; light gray rectangular boxes, dispersed tissue sites; dark gray filled box, heart. GI, gastrointestinal.

In addition to the blood within the organs, there is also blood in the large arteries (6%) and veins (18%) outside of the organs (9). This needs to be distributed across the parallel and entirely independent blood vessels of the CBGS model. Volumes for 128 segments of the major arteries were calculated from a detailed morphometric model of the arterial tree (14), as tablulated in the appendix (Fig. 2). These volumes were then rescaled to sum to 6% of total blood volume.

Following this, the 11 compartments were classified either as discrete tissues (heart, lungs, kidneys, liver, GI tract/spleen, and brain), where their specific arteries could be identified within the arterial tree, or else as dispersed tissues (muscle, skin, adipose, bone, and rest of body), where this was not the case. Conceptually, the discrete tissues were “attached” to their specific arteries. All other arteries were considered to supply the dispersed tissues. The number of dispersed tissue sites was progressively reduced by merging distal arteries with their attendant dispersed tissue until it was not possible to go further without merging one of the discrete tissues (Fig. 2). This resulted in 5 blocks of dispersed tissue: left and right head and neck supplied by the left and right external carotid arteries; left and right arms/torso supplied by the left and right subclavian arteries (distal to origin of vertebral arteries); and lower body supplied by the left and right iliac arteries.

The blood flow for the GI tract and spleen compartment arises from multiple arteries, and therefore the compartment’s total blood flow has to be apportioned between these. This apportioning was based on the blood flows to the large intestine [*vessel 16*, fraction of total perfusion (Qf) = 0.04], the small intestine (*vessel 15*, Qf = 0.1), and the stomach and spleen (*vessel 14*, Qf = 0.04) (9). In a similar manner, values for the resting blood flow to the arteries supplying the dispersed tissues were taken from the literature (23). Using these values, the following percent distributions for perfusion of the dispersed tissue sites were calculated: right head and neck (excluding brain) 3.7%, left head and neck (excluding brain) 3.3%, left and right arms/trunk 7.6%, and lower body 23.4%.

For all 128 segments of the arterial tree, the fraction of blood flow supplying each of the compartments of the CBGS model was calculated. This step allowed the blood volume for each segment of the arterial tree to be apportioned between the compartments of the CBGS model in proportion to blood flow. The total dispersed perfusion fraction that is split between these sites is equal to the sum of the dispersed tissues and the other tissues not specified in our model.

The ICRP data do not provide a break down for the different dispersed tissue types at the five sites of the model (left head, right head, left upper body, right upper body, and lower body). Consequently, these five sites were condensed into one single site per dispersed tissue type.

A volume for the portal vein was calculated from its diameter (17) and length (18). This was subtracted from blood volume in the large veins (9), and the remaining volume was distributed to the compartments in proportion to arterial blood volume. The results of these calculations are given in Table 1. The pulmonary blood volume was treated as a single compartment with blood entering from all compartments.

Metabolic rates for each tissue were taken from Ref. 24. The values in kcal/kg/day were converted to rates of oxygen consumption using a conversion factor of 4.825 kcal/L of oxygen consumed (25). Wang et al. (24) gave a residual value for metabolism per unit mass for the tissues not included in their study, and this was distributed across the remaining tissues of our model.

### Scaling the CBGS Model to Individuals

Individuals differ in size and body composition. To account for this, the model is scaled to a particular individual using their height, mass, sex, and age. First, an estimate of fat percentage by mass, *f_fat_*, is made using the following *Eq. 2*:

(*1*) $$\text{BMI}={\text{mass/height}}^{2}$$

(*2*) $$f_{\text{fat}}=\frac{1.2(\text{BMI})+0.23(\text{age})-10.8(isMale)-5.4}{100},$$

where BMI is body mass index, and *isMale* is set to 0 for women and 1 for men (26). This technique gives superior estimates of adiposity than BMI alone [Eq. *1* (27)].

An individual’s lean body mass can then be calculated by subtracting the mass of their adipose tissue from their total body mass. This enables an individual’s lean body mass and their adipose tissue mass each to be expressed as a fraction of that associated with the standard man. These fractions can then be used to produce an appropriately scaled model of the individual from the values associated with the standard man.

### Gas Exchange Between Circulation and Body Tissues

For inert gases, the equation for conservation of matter for each gas, *x*, across each compartment, *c*, is as follows:

(*3*) $$\left[V_{T}^{c}\beta_{T,x}^{c}+V_{b}^{c}\beta_{b,x}\right]\frac{dP_{v,x}^{c}}{dt}=Q^{c}\beta_{b,x}\left(P_{a,x}^{c}-P_{v,x}^{c}\right)$$

where $V_{T}^{c}$ is the tissue volume for compartment *c*; $\beta_{T,x}^{c}$ is the solubility for gas *x* in compartment *c*; $V_{b}^{c}$ is the volume of blood in the capillaries for compartment *c*; $\beta_{b,x}$ is the solubility for gas *x* in blood; $P_{v,x}^{c}$ is the partial pressure of gas *x* in the capillary/venous blood of compartment *c*; *t* is time; $Q^{c}$ is the perfusion to compartment *c*; and $P_{a,x}^{c}$ is the partial pressure of gas *x* in the arterial blood supplying compartment *c*.

The exchange for CO_2_ and O_2_ is coupled through the Bohr and Haldane effects, with conservation of matter across each compartment given as follows:

(*4*) $$\begin{array}{l} \left[\begin{array}{c} V_{T}^{c}\beta_{T,CO_{2}}^{c}+{V_{b}^{c}\frac{\partial C_{v,CO_{2}}^{c}}{\partial P_{v,CO_{2}}^{c}}|}_{P_{v,CO_{2}}^{c},P_{v,O_{2}}^{c}},{V_{b}^{c}\frac{\partial C_{v,CO_{2}}^{c}}{\partial P_{v,O_{2}}^{c}}|}_{P_{v,CO_{2}}^{c},P_{v,O_{2}}^{c}}\\ {V_{b}^{c}\frac{\partial C_{v,O_{2}}^{c}}{\partial P_{v,CO_{2}}^{c}}|}_{P_{v,CO_{2}}^{c},P_{v,O_{2}}^{c}},\;V_{T}^{c}\beta_{T,O_{2}}^{c}+{V_{b}^{c}\frac{\partial C_{v,O_{2}}^{c}}{\partial P_{v,O_{2}}^{c}}|}_{P_{v,CO_{2}}^{c},P_{v,O_{2}}^{c}} \end{array}\right]\left[\begin{array}{l} \frac{dP_{v,CO_{2}}^{c}}{dt}\\ \frac{dP_{v,O_{2}}^{c}}{dt} \end{array}\right]\\ \;\;\;\;\;\;\;\;\;=\left[\begin{array}{l} Q^{c}C_{a,CO_{2}}^{c}\left(P_{a,CO_{2}}^{c},P_{a,O_{2}}^{c}\right)-Q^{c}C_{v,CO_{2}}^{c}\left(P_{v,CO_{2}}^{c},P_{v,O_{2}}^{c}\right)+m_{CO_{2}}^{c}\\ Q^{c}C_{a,O_{2}}^{c}\left(P_{a,CO_{2}}^{c},P_{a,O_{2}}^{c}\right)-Q^{c}C_{v,O_{2}}^{c}\left(P_{v,CO_{2}}^{c},P_{v,O_{2}}^{c}\right)-m_{CO_{2}}^{c} \end{array}\right] \end{array}$$

where $C_{a,x}^{c}$ is the arterial concentration of gas *x* (here CO_2_ or O_2_) supplying compartment *c*; $C_{v,x}^{c}$ is the capillary/venous concentration of gas *x* leaving compartment *c*; and $m_{x}^{c}$ is the metabolic production or consumption for gas *x* in compartment *c*.

### Solubilities and Partition Coefficients

Both CO_2_ and O_2_ react reversibly with blood, where the total content of each gas (dissolved plus reversibly reacted) depends upon the partial pressures of both gases. We used the model of O’Neill and Robbins (28) to describe this relationship, which incorporates both the Bohr and Haldane effects. As well as dissolving physically in the tissues, CO_2_ also reversibly reacts with water to form H^+^ and HCO_3_^−^, and the extent to which this reaction occurs depends upon the intrinsic buffering of the cell type. Values for this buffering capacity are not reported for most cell types and so we adopted a value of 15 mmol/L/pH unit (29) with an intracellular water fraction of 70% as applicable for most tissues. For muscle, the value is higher, and we used the measured value of 25 mmol/L/pH unit reported for cardiac muscle (30). The intrinsic buffer capacities were converted to apparent CO_2_ solubilities using the Henderson Hasselbalch equation with an assumed intracellular pH of 7.2 (pH of 7.04 for cardiac muscle). These apparent solubilities were added to a physical solubility for CO_2_ in the tissue that was taken as equal to the value for plasma for all tissues but fat (31).

For O_2_, its ability to react reversibly with myoglobin in muscle was ignored as the model is only concerned with Po_2_ values where myoglobin is saturated with O_2_. For all tissues but fat, the intracellular solubility for oxygen was assumed to be equal to that in plasma.

Weathersey and Homer (31) have provided a review of inert gas solubilities in biological tissues and fluids. For setting the solubility of N_2_ in blood, we gave precedence to values for human blood and to values where the investigators had also reported solubilities in water that were close to their well-established values. For N_2_, the tissue solubilities were assumed to equal the blood solubility for all tissues except brain and fat. The solubility in the brain was taken as 6% higher than that for blood (31).

The adipose compartment required special treatment for all gases. For CO_2_, a physical solubility in human fat of 1.67 times its value in water has been reported (32). To obtain an overall solubility for adipose tissue, we assumed that the tissue was 80% fat and 20% aqueous (8), and that no proton buffering occurred in the fat element. For N_2_, the solubility in pure body fat has been reported to be around 5 times that in water (33). We also note that the partition coefficients between olive oil and water are very similar for N_2_ and O_2_ (at around 5). For this reason, to determine the O_2_ solubility in adipose tissue (80% fat, 20% aqueous) from its solubility in water, we used the same multiplier of 4.2 as we used for N_2_. The overall tissue solubilities used are given in Table 2 in terms of L_(STPD)_/L_(tissue)_/Pa.

**Table 2.** Gas-specific values for a standard man used in the circulation and body gas store model

Tissue	$m_{T,CO_{2}}$	$m_{T,O_{2}}$	$m_{T,N_{2}}$	*Ṁo_2_*
	$L_{\left(STPD\right)}L^{-1}Pa^{-1}$	$L_{\left(STPD\right)}L^{-1}Pa^{-1}$	$L_{\left(STPD\right)}L^{-1}Pa^{-1}$	$L_{\left(STPD\right)}L^{-1}s^{-1}$
Adipose	1.05 × 10${}^{-5}$	10.78 × 10${}^{-7}$	5.67 × 10${}^{-7}$	1.08 × 10${}^{-5}$
Bone	1.54 × 10${}^{-5}$	2.27 × 10${}^{-7}$	1.28 × 10${}^{-7}$	2.88 × 10${}^{-5}$
Brain	1.54 × 10${}^{-5}$	2.27 × 10${}^{-7}$	1.35 × 10${}^{-7}$	57.57 × 10${}^{-5}$
GI tract	1.54 × 10${}^{-5}$	2.27 × 10${}^{-7}$	1.28 × 10${}^{-7}$	2.88 × 10${}^{-5}$
Heart	1.70 × 10${}^{-5}$	2.27 × 10${}^{-7}$	1.28 × 10${}^{-7}$	105.55 × 10${}^{-5}$
Kidneys	1.54 × 10${}^{-5}$	2.27 × 10${}^{-7}$	1.28 × 10${}^{-7}$	105.55 × 10${}^{-5}$
Liver	1.54 × 10${}^{-5}$	2.27 × 10${}^{-7}$	1.28 × 10${}^{-7}$	47.98 × 10${}^{-5}$
Lungs	1.54 × 10${}^{-5}$	2.27 × 10${}^{-7}$	1.28 × 10${}^{-7}$	2.88 × 10${}^{-5}$
Muscle	1.70 × 10${}^{-5}$	2.27 × 10${}^{-7}$	1.28 × 10${}^{-7}$	3.12 × 10${}^{-5}$
Skin	1.54 × 10${}^{-5}$	2.27 × 10${}^{-7}$	1.35 × 10${}^{-7}$	2.88 × 10${}^{-5}$
Blood	*	*	1.44 × 10${}^{-7}$	
Rest of body	1.54 × 10${}^{-5}$	2.27 × 10${}^{-7}$	1.28 × 10${}^{-7}$	2.88 × 10${}^{-5}$
Citation	(1, 6)	(34)	(3, 35–37)	(24, 25)

$m_{T,X}$, tissue gas dissociation gradient for gas *X*; *Ṁo_2_*, oxygen consumption; *, not constant and the values arise from the blood gas dissociation curves. At a standard mixed venous $P_{\text{C}{\text{O}}_{2}}$ of 6.1 kPa and $P_{{\text{O}}_{2}}$ of 5.3 kPa the values are approximately 3.12 × 10${}^{-5}$ $L_{\left(STPD\right)}L^{-1}Pa^{-1}$ for CO_2_ and 2.26 × 10${}^{-5}$ $L_{\left(STPD\right)}L^{-1}Pa^{-1}$ for O_2_, where STPD refers to standard temperature and pressure, dry.

### Initial Conditions

To initialize the model under steady-state conditions, it is sufficient to know arterial blood gas contents for CO_2_, O_2_, and N_2_. This blood fills the arterial tree, and the metabolic exchange and blood flow to the individual tissues determines the tissue stores and venous blood concentrations for each compartment. If the mixed venous rather than the arterial concentration is known, the arterial concentration is easily calculated from the cardiac output and the total metabolic oxygen consumption and carbon dioxide production of the tissues.

If it is desirable to depart from either the standard metabolic rate or cardiac output specified by the model for an individual, it is necessary to apportion these variations across the tissues of the model. In the case of moderate exercise, it is reasonable to focus any increase in blood flow or metabolism on skeletal muscle. (In heavy exercise, there will be some reductions in the perfusion of other organs.) Variations in metabolic rate at rest are likely to arise predominately from fluctuations relating to the digestion, absorption, and metabolism of food. Although somewhat arbitrary, we chose to ascribe 60% of any variation in metabolic rate to the GI tract, liver and skeletal muscle, and apportion the remaining 40% across all other tissues in relation to their existing metabolism. As blood flow tends to follow metabolism, we used the same approach for distributing variations in cardiac output.

### Implementation

The model was written in Matlab and will run on a standard Windows (or other) desktop. The circulation is first established as an object. The model input is an array, where the columns contain the cumulative blood volumes (based on the blood flow) and gas contents. Each row reflects an aliquot of blood and we chose to work with aliquots based on a fixed 10 ms step size for the simulation. The output is an array of the same dimensions supplying the cumulative venous outflow volumes (which are the same as for the input) and the cumulative venous gas contents. If it is desired to run the model as a closed loop circulation, the model can be interfaced with any lung model that is capable of supplying the pulmonary venous outflow and receiving the pulmonary arterial inflow in the form described. The venous output can be mixed back into a pulmonary blood volume, if required.

### CBGS Execution

During execution, the blood is passed into the segments of the arterial tree in proportion to their share of total blood flow. For each compartment, an equivalent amount of blood is drawn from the distal end of the arterial tree; this enters the capillary bed for the compartment and displaces an equal volume of blood into the associated vein. The new capillary blood is mixed with the remaining capillary blood and then the gases are equilibrated between blood and tissue according to *Eqs. 3* and *4*. Finally, the blood added to the veins in each compartment is used to displace equal volumes from the central ends of the veins, and is then mixed with the pulmonary blood volume. Using an array with 100 columns (equivalent to one second of blood flow), the model runs 20 times faster than real time. Thus it takes 3 minutes to simulate one hour of circulation.

### Model Validation

An ideal approach to model validation would be to vary the alveolar gas composition in a set of volunteers and then to measure sequentially the composition of the mixed venous blood as it returns to the lungs. These sequential measurements could then be compared with those predicted by the model. However, the measurement of mixed venous composition is an invasive procedure that requires a catheter in the pulmonary artery, and this is difficult to justify ethically in healthy volunteers. As a result, model validation has proceeded by simulating experiments from the literature to determine whether the model can predict their reported results and by undertaking experimental studies where the output variable is respired gas exchange rather than mixed venous content.

### Simulation of Previously Reported Studies

In the case of N_2_, Groom et al. (38) provided experimental data for the mixed venous concentration of nitrogen in dogs at two time points following the introduction of pure oxygen in the inspirate. These values could be compared with those obtained by simulating the introduction of pure oxygen into a lung using our model. In addition, Baker and Farmery (3) provided a simple four-compartment model for nitrogen stores in humans with which our results may be compared.

With respect to CO_2_ carriage and storage, there are a number of reports describing the rate of increase of arterial Pco_2_ that occurs during anesthesia under conditions of apneic oxygenation, with sequential values reported (39). This rise is predominantly determined by the storage of CO_2_ in the tissues and can be simulated by our model. In this case the values for cardiac output and metabolism in the CBGS model were adjusted to match those for an average human under anesthesia (40, 41). Initial gas concentrations were set by equilibrating the arterial blood with high oxygen, while keeping CO_2_ at normal levels (39). A simplified homogeneous lung, with alveolar Pco_2_ and Po_2_ to match the arterial concentrations, was then sealed at a supine functional residual capacity (FRC) lung volume of 2.2 L (42). The time course for the rise in the systemic arterial Pco_2_ was computed from the model, and this could then be compared with the experimental results.

We were unable to identify any suitable data from the literature with which to validate the oxygen stores of our model.

### Experimental Validation

All experimental work on human participants was carried out in accordance with the general principles of the Declaration of Helsinki, with ethical approval obtained from the South Central Oxford A Research Ethics Committee (reference number 17/SC/0172). All participants provided written informed consent before the study.

For each validation experiment, three participants were studied. In the case of the oxygen validation experiment, one of these participants was not available and another was therefore substituted. The physical characteristics of the participants are given in Table 3. Participants were asked to refrain from food two hours prior to study to avoid post-prandial changes in metabolic rate and acid-base balance. Experimental studies were conducted using a molecular flow sensing device to provide highly accurate, highly precise measurements of gas exchange (43). In brief, the device uses laser absorption spectroscopy to measure CO_2_, O_2_, and water vapor across the mainstream respired gas flow every 10 ms. Nitrogen is calculated as the balance gas after CO_2_, O_2_, and water vapor have been determined. The simultaneous measurement of these gases, together with measurements of absolute temperature and pressure, enables the viscosity and density of the gas mixture to be calculated, and these in turn enable a highly precise measurement of instantaneous gas flow to be made.

**Table 3.** Participant characteristics

Participant	Sex	Height /m	Weight /kg	Age /yr	FRC /$L_{\left(BTPS\right)}$	A-a O_2_ gradient /kPa
#1	M	1.85	80	25	4.63	1.23
#2	M	1.79	90	39	2.08	0.78
#3	M	1.84	71	34	4.49	1.00
#4	F	1.65	57.5	22	2.33	0.69

M, male; F, female; FRC, function residual capacity; A-a O_2_ gradient, alveolar-arterial O_2_ gradient; BTPS, body temperature and pressure, saturated.

To compare the experimental results for gas exchange at the mouth with the model output, a model for the lung has to be incorporated between mixed venous outflow and systemic arterial inflow to the CBGS model. This lung model can be driven by the experimentally recorded inspiratory and expiratory flows together with the inspired gas composition. The outputs of the model are the N_2_, CO_2_, and O_2_ concentrations within the total expiratory flow, and these enable the breath-by-breath gas exchange for the model to be calculated.

The particular model of the lung used was that of Mountain et al. (44). This model allows for a number of inhomogeneities to be present. Importantly, participant-specific values for these lung parameters could be estimated in preliminary experiments according to the washout protocol of Mountain et al. (44). The alveolar-arterial gradient for O_2_ from the model over the air-breathing period is shown in Table 3 for each participant. For each of the validation experiments performed, oxygen consumption and carbon dioxide production were estimated from the preliminary air breathing phase of the validation experiments. These metabolic rates were incorporated into the CBGS model. This left just the initial conditions to be estimated from the validation experiments, which were the starting concentrations for the gases in the systemic venous blood and tissues together with the starting gas concentrations and volumes in the lung.

To explore the performance of the CBGS specifically, exactly the same process as that outlined above was followed but with a dummy CBGS model in place which contained no dissolved or reacted gas stores.

No experimental validation was attempted for N_2_ because the contribution from the lung gas stores associated with any change in inspired N_2_ would swamp the exchange with blood until a new steady state for lung gas stores was established, and after that period the magnitude of the N_2_ exchange with the blood would be too close to the limits of precision for the molecular flow sensor (even for a complete washout of N_2_ with pure O_2_) (43).

Two validation experiments were conducted for CO_2_. The first of these involved increasing CO_2_ to above normal by rebreathing gas contained within a 6 liter anesthetic bag. The bag was filled to a volume of ∼4 liters with a mix of O_2_ and CO_2_ such that the CO_2_ partial pressure matched the mean end-tidal CO_2_ measured during the air phase of the Mountain et al. (44) protocol. The experiment began with the participant breathing room air for 5 min, after which rebreathing was started at the end of expiration, at FRC, by switching the inspirate from air to rebreathing from the bag.

The second validation experiment for CO_2_ involved decreasing the CO_2_ to below normal by asking the participant to undertake a period of voluntary hyperventilation. The participant breathed normally for 5 min and then was asked to increase their tidal volume and breathing frequency to target an end-tidal partial pressure for CO_2_ of 3.33 kPa.

A single validation experiment was performed for the oxygen stores. As the primary store of oxygen is that bound to hemoglobin in the circulating blood, and as the arterial blood is close to fully saturated with oxygen during air breathing, the required experiment is one that reduces the oxygen saturation of hemoglobin in the blood. Therefore, the protocol involved reducing the oxygen in the lungs by washing it out with a hypoxic gas mixture. The protocol started with the participant breathing room air for 5 min. The inspired gas was then switched to pure nitrogen for 2–3 breaths before being changed to a mixture containing oxygen at ∼ 10 kPa for ∼ 3–4 min. A pulse oximeter was used to check that arterial saturation remained above 80%. The participant was returned to breathing air for the final ∼ 3 to 4 min of the protocol.

## RESULTS

**Figure 3.**
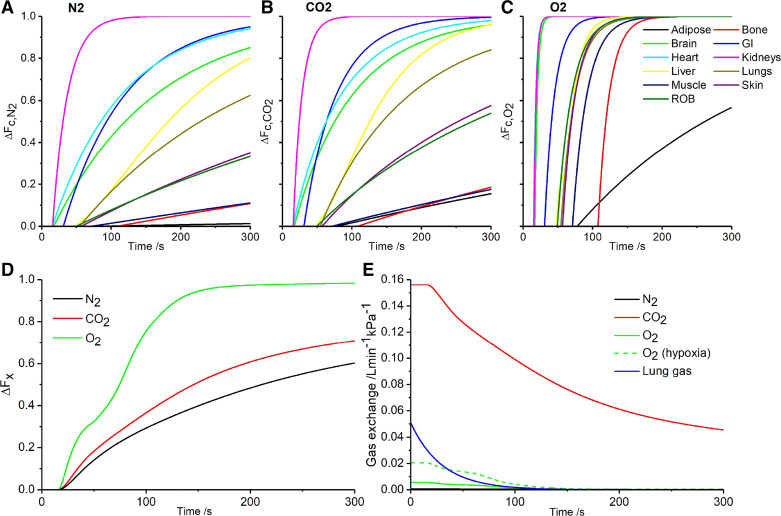
The responses of the circulatory model to a step change in 1 kPa for N_2_, CO_2_, and O_2_. *A-C*: fractional change from old to new steady-state venous concentrations for each of the 11 compartments after a 1 kPa rise in N_2_, Δ*F_c,N_*_2_ (*A*); CO_2_, Δ*F_c,CO_*_2_ (*B*); and O_2_, Δ*F_c,O_*_2_ (*C*). *D*: fractional change from old to new steady-state concentration in the pulmonary vascular blood volume after a 1 kPa rise for each of the gases, Δ*F_x_*. *E*: variation in gas exchange at the level of the pulmonary capillaries for each of the three gases following a 1 kPa step increase in alveolar/arterial gas tension. Also shown are *1*) the variation in gas exchange for a change in alveolar/arterial O_2_ from 6.66 to 13.3 kPa and *2*) the variation in gas exchange at the mouth for a 1 kPa change in alveolar concentration. Parameters used for generating plots are those for the standard man. ROB, rest of body.

The overall properties of the model were first explored by imposing a 1 kPa step increase in partial pressure at the arterial inflow for each of three gases in turn and plotting the subsequent changes in venous blood composition (Fig. 3). Fig. 3, *A–C*, illustrates the change in concentration (as a fractional change toward the new steady-state value) at the venous outflow for the individual compartments of the model for N_2_, CO_2_, and O_2_, respectively. The different delays before the onset of the response are apparent on the *x*-axis and these reflect the different circulatory times for the different compartments. It is clear that, once any given transient perturbation has begun, the subsequent speed of equilibration differs substantially between the tissues. The fastest compartment is renal, which is unsurprising since the kidneys receive ∼20% of the total cardiac output but have a combined mass of only ∼300 g.

From Fig. 3, *A-C*, it is also apparent that the speed of response between the different tissues is both faster and far less varied for O_2_ than it is for CO_2_ or N_2_. This arises because, even in euoxia, the ability of hemoglobin to reversibly bind O_2_ dominates the solubility of O_2_ in the body. The speeds of response appear somewhat slower for N_2_ than for CO_2_, and this arises because, unlike N_2_, the apparent solubility of CO_2_ in the blood is somewhat greater than the apparent solubility of CO_2_ in the tissues (Table 2). The slowest response is for N_2_ for the adipose tissue compartment, where equilibration has hardly begun at the 5 min point. This arises because N_2_ is substantially more soluble in fat than it is in an aqueous environment.

Fig. 3*D* illustrates the fractional change toward the new steady-state concentrations in the mixed venous blood for N_2_, CO_2_, and O_2_. Unsurprisingly, the fastest change is for O_2_. At the 5 min time point, the concentrations for N_2_ and CO_2_ are both more than 50% toward their new equilibrium values. These fractional changes in venous concentration can be very different from the fractional completion for the change in the amount of gas stored. Indeed, in the case of N_2_, more than half of the gas store is predicted to be in the adipose tissue compartment, and that has barely begun to change at the 5 min time point.

Fig. 3*E* illustrates the rate at which N_2_, CO_2_, and O_2_ are being loaded into the body stores via the circulation (these values would be zero for each gas under equilibrium conditions) for the 1 kPa increment in partial pressure in the arterial inflow. For purposes of comparison, Fig. 3*E* also illustrates the rate at which a gas would be loaded into the gas stores of the lung for a 1 kPa increment in partial pressure in the inspired gas. The rate of loading of CO_2_ vastly exceeds the rate of loading of the other two gases while the rate of loading for N_2_ is scarcely visible above the *x*-axis. For N_2_, the comparison of exchange rates between the lung gas stores and the tissue gas stores illustrates why it was not possible to undertake validation experiments through measuring N_2_ exchange at the mouth. For O_2_, the exchange can be enhanced by incorporating a degree of hypoxia so that the storage is operating over the steep part of the oxygen dissociation curve for blood. Fig. 3*E* illustrates this as a mean rate of oxygen storage per kPa over approximately the range of Po_2_ employed in the validation experiments.

### Nitrogen Validation

**Figure 4.**
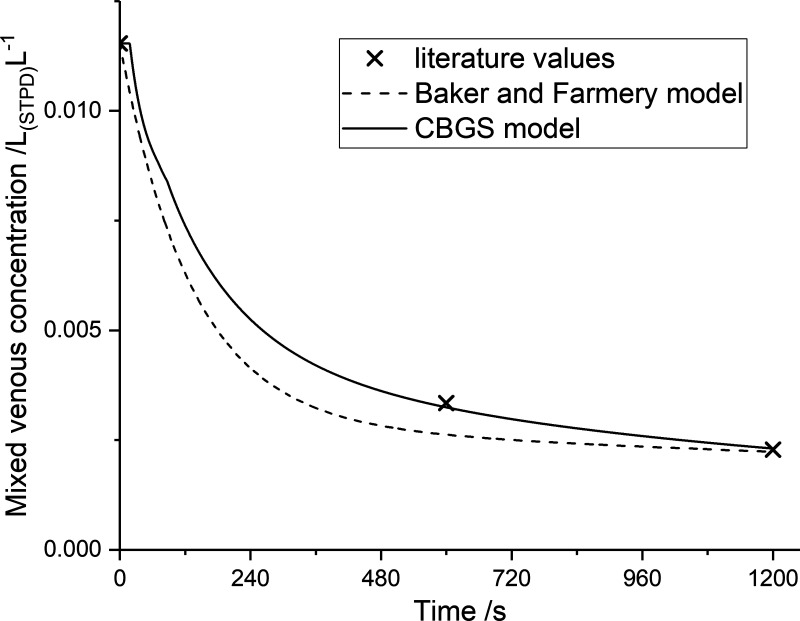
Mixed venous N_2_ concentration following a switch to breathing pure oxygen. Full line, CBGS simulation (parameters are as for standard man); broken line, model of Baker and Farmery (3); symbols, measured values for dog (25). CBGS, circulation and body gas stores.

As previously indicated, it was not possible to undertake experimental validation of the model for nitrogen exchange. A comparison between a simulated nitrogen washout using the CBGS model could be drawn with both experimental data from the dog (38) and with a prior model of nitrogen stores given by Baker and Farmery (3). This comparison is shown in Fig. 4. The model predicted the experimental data points for the concentration of nitrogen in the mixed venous blood extremely well. The CBGS model was also consistent with the Baker and Farmery (3) model at the later time points, but it gave concentrations for nitrogen that were significantly higher at earlier time points. In relationship to this, it should be noted that the Baker and Farmery (3) model is a simple four-compartment model with no circulatory lags.

### Carbon Dioxide Validation

**Figure 5.**
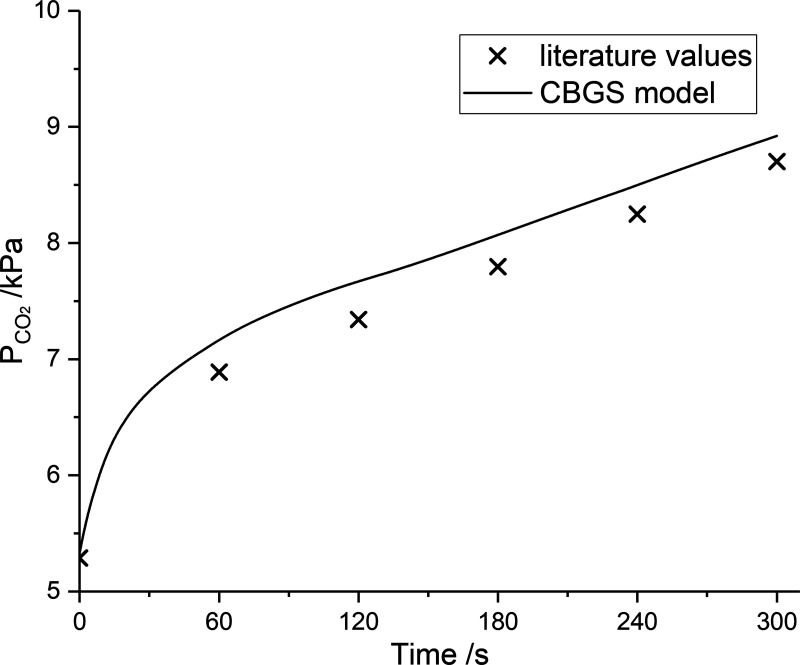
Increase in arterial Pco_2_ during a period of tracheal clamping under conditions of pure oxygen. Full line, CBGS simulation; symbols, data from an anesthetized human (50). Circulation and body gas stores (CBGS) parameters were for the standard man but with a metabolic rate appropriate for anesthesia.

The literature provides data for the change in arterial partial pressure for CO_2_ over time in anesthetized patients who have first been given pure oxygen to breathe and then had their trachea occluded (39). Typically, the arterial partial pressure for CO_2_ rises by ∼1.6 kPa in the first minute and this is followed by an increase of ∼0.45 kPa/min for the next ∼4 min (39). Fig. 5 illustrates these values together with a simulation of the experiment using the CBGS model. Although there is some difference in the absolute values between data and model, the agreement between model and data for the overall form of the response is extremely good. The model gradient is 1.8 kPa/min in the first minute followed by 0.44 kPa/min for the next 4 min. It is of note that 92% of the CO_2_ produced in this experiment is stored in the tissues and only some 8% in gaseous form in the lung.

**Figure 6.**
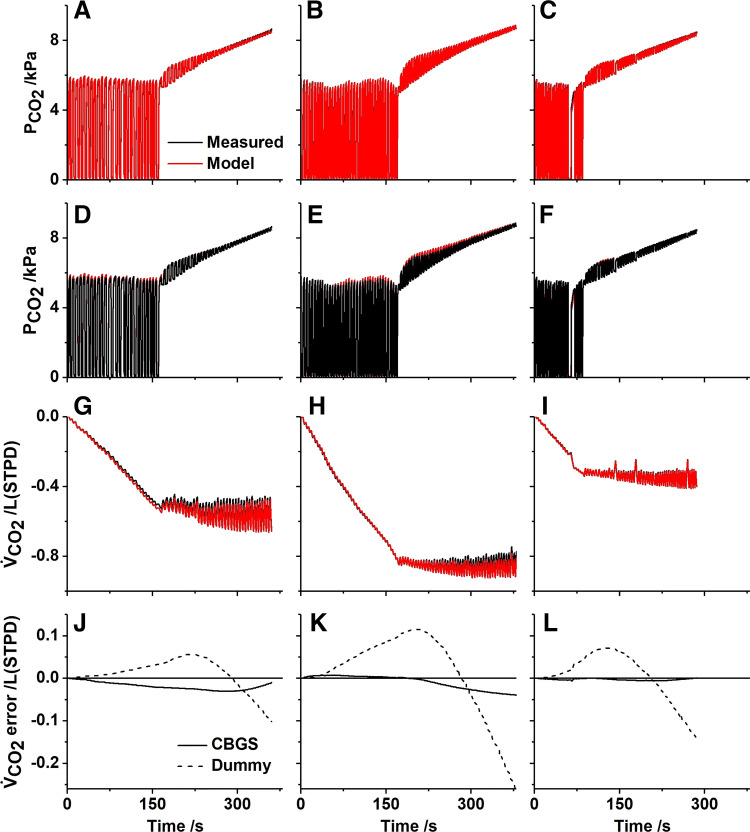
Experimental and model responses to rebreathing from a 6 liter bag for three participants. *A-C*: airway Pco_2_, model superimposed on data. *D-F*: airway Pco_2_, data superimposed on model. *G-I*: cumulative CO_2_ production (V̇co_2_), model superimposed on data. *J-L*: cumulative error in CO_2_ production (model - measured) when the CBGS model is included (full line) and when a dummy circulation is used (broken line). *A, D, G*, and *J: participant 2*. *B, E, H*, and *K*: *participant 3*. *C, F, I*, and *L*: *participant 4*. To derive the model output, participant-specific inhomogeneity parameters were taken from a prior experiment; O_2_ consumption and CO_2_ production were estimated from the air breathing phase of the experiment; and the initial mixed venous values together with the alveolar lung volume were estimated from the data. CBGS, circulation and body gas stores.

Experimentally, rebreathing was studied in three subjects using an anesthetic bag prefilled with a mixture of carbon dioxide, at a partial pressure designed to match the alveolar Pco_2_ (∼5.5 kPa), and pure oxygen. The comparison between the experimental results and model simulations is shown in Fig. 6. Both the CO_2_ profiles in the airway and the records for the cumulative production of CO_2_ are very similar between model and data. The cumulative error is always less than 5% of the total exchange. For comparison, the error for the cumulative CO_2_ exchange is also shown in the case where the CBGS model has been replaced with a dummy model which does not contain any gas stores. Here, the cumulative errors in exchange are far greater, and Table 4 demonstrates that the mean squared error for the breath-by-breath CO_2_ exchange is between 16- and 56-fold greater for the dummy model as compared with the CBGS model.

**Table 4.** Quality of fit with and without circulatory model

Hyperventilation		
Participant	$\varepsilon_{CO_{2}}$ /${\left(Lmin^{-1}\right)}^{2}$	$\varepsilon_{\text{C}{\text{O}}_{2}}^{\mathrm{CBGS}}$ /${\left(Lmin^{-1}\right)}^{2}$
#2	0.0068	0.0019
#3	0.0129	0.0007
#4	0.0081	0.0004

*Rebreathing*		
Participant	$\varepsilon_{CO_{2}}$ /${\left(Lmin^{-1}\right)}^{2}$	$\varepsilon_{C{\text{O}}_{2}}^{\mathrm{CBGS}}$ /${\left(Lmin^{-1}\right)}^{2}$
#2	0.0059	0.0004
#3	0.0232	0.0004
#4	0.0126	0.0005

*Oxygen washout*		
Participant	$\varepsilon_{O_{2}}$ /${\left(Lmin^{-1}\right)}^{2}$	$\varepsilon_{O_{2}}^{\mathrm{CBGS}}$ /${\left(Lmin^{-1}\right)}^{2}$
#1	0.0083	0.0053
#2	0.0031	0.0020
#3	0.0018	0.0008

$\varepsilon_{x}$, mean squared error between the measured and simulated rate of exchange of gas *x* at the mouth without the model for the circulatory and body gas stores; $\varepsilon_{x}^{\mathrm{CBGS}}$, mean squared error between the measured and simulated rate of exchange of gas *x* at the mouth with the model for the circulatory and body gas stores.

**Figure 7.**
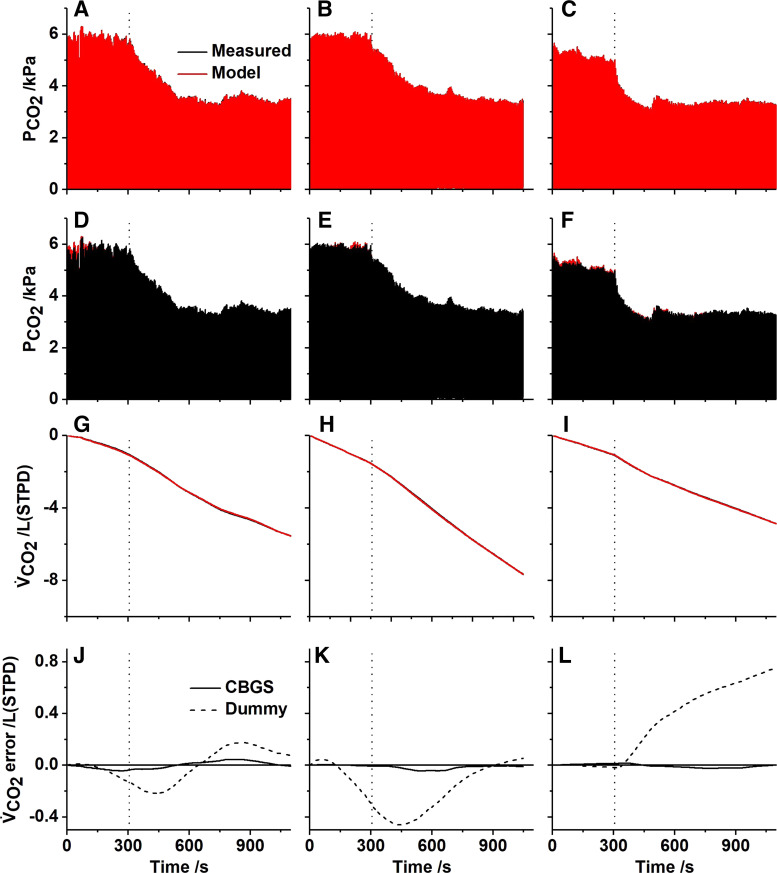
Experimental and model responses to voluntary, paced hyperventilation for three participants. *A-C*: airway Pco_2_, model superimposed on data. *D-F*: airway Pco_2_, data superimposed on model. *G-I*: cumulative CO_2_ production (V̇co_2_), model superimposed on data. *J-L*: cumulative error in CO_2_ production (model - measured) when the CBGS model is included (full line) and when a dummy circulation is used (broken line). *A, D, G*, and *J*: *participant 2*. *B, E, H*, and *K*: *participant 3*. *C, F, I*, and *L*: *participant 4*. To derive the model output, participant-specific inhomogeneity parameters were taken from a prior experiment; O_2_ consumption and CO_2_ production were estimated from the air breathing phase of the experiment; and the initial mixed venous values together with the alveolar lung volume were estimated from the data. CBGS, circulation and body gas stores.

To study unloading the body stores of CO_2_, a further set of experiments were conducted during which the participants undertook a period of voluntary hyperventilation. The comparisons between the experimental results and model simulations are shown in Fig. 7. As for the rebreathing experiments, both the airway partial pressures and cumulative gas exchange records for CO_2_ were very similar between model and data. The error in the cumulative CO_2_ production between data and model is shown for both the CBGS model and the dummy model, and this illustrates clearly the importance of the body gas stores. This is confirmed in Table 4, where the mean squared error for the breath-by-breath CO_2_ exchange is between 4- and 22-fold greater for the dummy model compared with the CBGS model.

### Oxygen Validation

**Figure 8.**
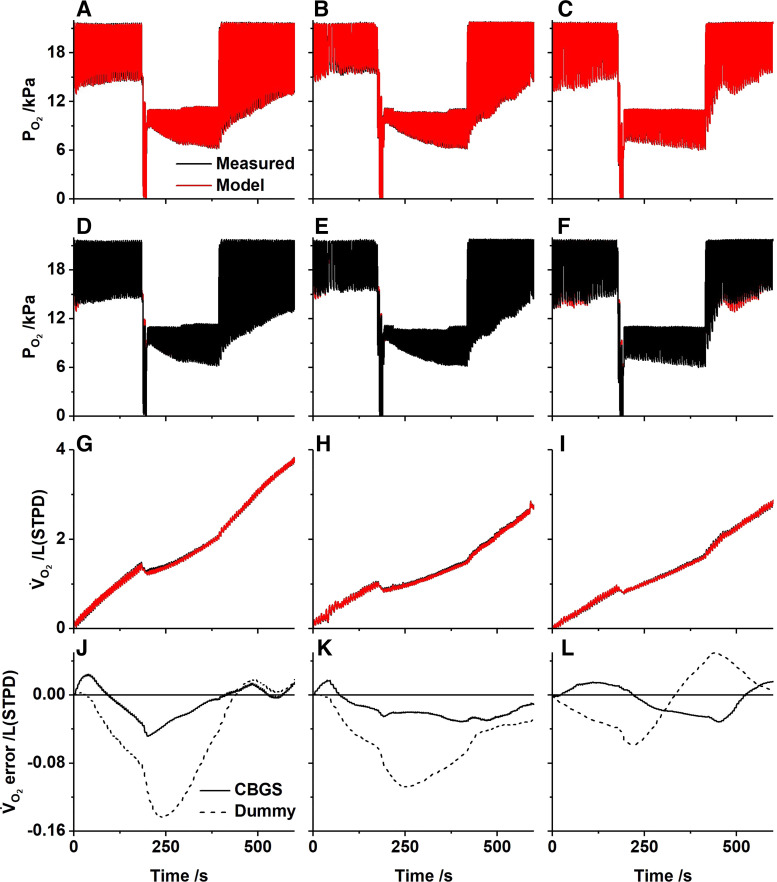
Experimental and model responses to the nitrogen washin protocol for three participants. *A-C*: airway Pco_2_, model superimposed on data. *D-F*: airway Po_2_, data superimposed on model. *G-I*: cumulative O_2_ production (V̇co_2_), model superimposed on data. *J-L*: cumulative error in O_2_ consumption (model - measured) when the CBGS model is included (full line) and when a dummy circulation is used (broken line). *A, D, G*, and *J*: *participant 1*. *B, E, H*, and *K*: *participant 2*. *C, F, I*, and *L*: *participant 3*. To derive the model output, participant-specific inhomogeneity parameters were taken from a prior experiment; O_2_ consumption and CO_2_ production were estimated from the air breathing phase of the experiment; and the initial mixed venous values together with the alveolar lung volume were estimated from the data. CBGS, circulation and body gas stores.

For oxygen, there were no data in the literature with which the model simulations could be compared. However, it was possible to reduce the oxygen stores somewhat by lowering the inspired oxygen to generate an element of desaturation in the arterial blood. The results are shown in Fig. 8. The profiles for the experimental and model airway Po_2_ values were very similar for all participants, as were the cumulative records for the O_2_ uptake. The cumulative error between data and model is shown for both the CBGS and dummy models. Again, these errors were clearly smaller for the CBGS model. In this case, the mean squared error for the breath-by-breath O_2_ exchange is at least 50% greater for the dummy model compared with the CBGS model for all participants (Table 4).

## DISCUSSION

The general purpose of developing the CBGS model was to provide a means to convert open system models for studies of gas exchange in the lung to closed system models, where the composition of blood leaving the lungs can subsequently influence the composition of blood returning to the lungs. In particular, we sought to develop a model that was related to anatomy and tissue type, that could be scaled to overall body size, that was relevant to both the respiratory gases and inert gases, and that included circulatory transit times as well as tissue storage capacities.

There is an immense amount of detail that relates to the circulation of blood through the body. It supplies many different organs via a complicated branching network for both the arterial and venous trees. The approach adopted in this work was pragmatic and was to focus on just the major organs and the anatomy of the major arteries and veins. Ultimately, it rests with validation studies to determine whether the model is sufficiently realistic for its purpose or whether additional detail or refinement is required.

The ICRP (9) provided a useful starting point for tissue masses, together with their blood contents and blood flows. Furthermore, the tissue solubilities for nitrogen and oxygen are fairly well characterized. The same is not true for carbon dioxide, where early attempts to measure tissue storage capacities gave rather variable results that depended on equilibration time (6). Carbon dioxide reacts with water to form bicarbonate and protons. The latter can be buffered intracellularly, and this in turn is likely to give rise to transmembrane ion fluxes that may underlie the somewhat variable results. Indeed, in relationship to intracellular buffering, Roos and Boron (45) wrote “Thus estimates [of buffering power] that are contaminated by ion fluxes lack definition and have meaning only when the experimental conditions are carefully defined.” For this reason, we chose to work from estimates of intrinsic buffering power where such potential shifts had been minimized. Of course, in vivo such ion fluxes will begin even over relatively short timescales including over minutes such as in our investigations. One possibility would have been to adopt or develop a detailed model of exchange between the intracellular and interstitial compartments and between the interstitial compartments and blood, such as those by Rees and Andreassen (46) and Wolff (47). However, many of the parameters required for such models are not well described, and over the relatively short timescales with which our model is concerned (∼10 min), these ion shifts tend more to redistribute the “site” of CO_2_ storage, rather than fundamentally change the overall carbon dioxide storage capacity. Over longer time scales (∼hours), slower processes like CO_2_ storage within bone matrix or bicarbonate excretion by the kidney become more important and these will be affected by such transmembrane ion fluxes. However, these aspects of CO_2_ storage are not very significant over the relatively short time courses with which we are concerned.

The model has been designed so that it can be used with inert gases other than nitrogen provided that the relevant solubilities are known. These have been collated for a number of the more common gases (31). Where these are not known, one possible approach is to use a theoretical calculation of the tissue partition coefficients, such as that developed by Abraham et al. (48, 49). However, for a few cases where the experimental values were known, we found that the theoretical calculation was not very precise, and indeed it could be better just to assume that the blood-tissue partition coefficient was unity.

The model of Ref. 14 provided the starting point for the arterial tree. One of the tasks in developing our model was to find an appropriate way to convert this branching model into the simpler system of a set of independent, parallel circulations to the chosen tissue types of the model. One unavoidable inaccuracy of this approach is the loss of an interdependence in the blood flows, whereby an increase in blood flow to one tissue will also reduce the circulation times for other tissues which have large arteries and veins in common. In retrospect, it would have been possible to maintain a branching structure for the very largest arteries and veins. Fortunately, the blood volumes and therefore transit delays for these are not so very large, e.g., 3.5% for the aorta.

Another approximation of the model is the condensing of the distributed tissues into single compartments, where the reality is that the circulatory lag times will vary substantially with actual anatomic location. Indeed, even with the discrete tissues there will be inhomogeneity in blood flow within them, for example, between the cortex and medulla of the kidney. Some of these approximations will matter more than others, and future studies could approach this by conducting sensitivity analyses to see how important they are for different scenarios and gases.

An ideal route to validation of the present model would have been to introduce, via the gases in the lung, known systemic arterial concentrations and then follow the changes in mixed venous concentrations over time. However, this would have required the insertion of a pulmonary arterial catheter and this could not be justified in healthy volunteers. Instead, the validation had to proceed either by attempting to predict existing findings within the literature or by conducting experiments and measuring instead the gas exchange at the mouth. Measurement of gas exchange at the mouth is far less satisfactory than measurement of mixed venous concentrations because changes in the lung stores for the gases have the potential substantially to affect the results obtained. Furthermore, it becomes necessary to incorporate a computational model of the lung between the venous and arterial ends of the CBGS model to allow for the changes in lung gas stores. To demonstrate that the CBGS model was necessary to model the gas exchange accurately, the process was also followed using a dummy CBGS model that had no body gas storage capacity. For both CO_2_ and O_2_, for all participants, the fit to the gas exchange data was very substantially worse with the dummy CBGS model in place.

The challenge of conducting the validation through measurements of gas exchange at the mouth is at its greatest for the least soluble gases. These have the smallest storage capacities in the compartments of the CBGS model, and gas exchange at the mouth is completely dominated by the lung gas stores. In this sense, the validation will have been more reliable for CO_2_ than for O_2_. In the case of N_2_, experimental validation through measurements of gas exchange at the mouth was not feasible for this reason. Thus, at the current time the validation of the model for inert gases is distinctly limited. One potential way forward for validating the model further for inert gases is to use tracer gases that have far greater solubilities than N_2_, such as acetylene and nitrous oxide. Indeed, these gases have been used for estimating cardiac output from the rate of their uptake from the lung. During such experiments, it would also be valuable to explore the influence of different body builds in greater detail.

The CBGS model can be compared with previous models for the body’s gas stores, and for N_2_ we have illustrated the comparison with that of Ref. 3 in Fig. 4. Here, the only experimental data are for the dog, and therefore it is only possible to note that the models differ somewhat during the initial, more rapid transient but converge at later time points.

Several models of body CO_2_ storage have been reported in the literature. Single-compartment models reproduce experimental data very poorly (6). Some multi-compartment models perform better, and over time periods longer than ∼30 min can closely reproduce experimental data (1, 2). However, they reproduce poorly the effect of CO_2_ storage in shorter experiments. Their fit to the data is only improved with post hoc manipulations such as unrealistic redistributions in perfusion, unrealistic changes in metabolism or arbitrary changes in the tissue CO_2_ solubility in a subset of tissues to match that of water (50). In contrast, our model reproduces experimental perturbations in CO_2_ very well across these shorter time frames without any physiologically unrealistic manipulations.

For whole body O_2_ stores, the only dynamic model of which we are aware is that of Cherniack and Longobardo (51). They incorporated O_2_ solubilities into the CO_2_ model of Farhi and Rahn (1). This model reproduced experimental perturbations in body O_2_ stores over a 3-min period poorly unless O_2_ consumption was reduced at a specific time point. While such a model summarizes the specific experiment, it clearly cannot be used to predict responses more generally for other scenarios. In contrast, our model tracked the dynamic fall and rise in O_2_ stores over an ∼10-min protocol without the need to postulate adaptive changes in metabolism.

The CBGS model has a number of potential applications, especially when coupled with a model of the lung such as that provided by Mountain et al. (44) and as used in the experimental validation. Importantly for these applications, both the CBGS and LNL models can be tailored to a particular body size and makeup so that it is possible to accommodate variations between individuals. One application relates to the study of stability of respiratory control, a topic of interest to high altitude physiology and to medical conditions such as heart failure and central or complex sleep apnea (52). Central to understanding stability in an individual is the measurement of loop gain. One-half of this loop relates to controller sensitivity, essentially how ventilation changes as arterial Pco_2_ and Po_2_ are varied, and for which there are many dynamic models and estimation processes for tailoring them to individuals, e.g., see Ref. 53. The second half of the loop relates to the plant sensitivity, which is how arterial Pco_2_ and Po_2_ change as ventilation is varied. Here there are far fewer models, and the present study helps to fill this gap. Importantly, our model can both be tailored to specific individuals and integrates both CO_2_ and O_2_ stores enabling a single loop gain to be calculated as both of these respiratory stimuli vary simultaneously.

A second potential application is in multi-breath washout testing. Multi-breath washout tests are increasingly being used in clinical studies to delineate the lung physiology of individual patients (54). In these studies, it is generally the case that no correction is made for the amounts of gas entering and leaving the lung through the pulmonary circulation. This is a significant limitation, as the results have been shown to depend on the solubility of the test gas (55), and in the case of a nitrogen washout that is accomplished by breathing pure oxygen, the results are also likely to be affected by changes in oxygen uptake. The CBGS model provides a potential way to correct for these effects.

A third potential application relates to improving respiratory techniques for determining cardiac output. These techniques, as reviewed by Laszlo (56), typically involve the use of a soluble tracer gas such as nitrous oxide or acetylene and measuring the rate of its disappearance from the lungs. While the subsequent calculation of cardiac output has the advantage of being firmly rooted in mass balance, it nevertheless assumes no recirculation of the tracer gas back to the lungs. The CBGS model suggests a possible approach whereby recirculation could be taken into account in the estimation of cardiac output by these methods, so making the calculation more accurate and also offering the possibility of allowing a somewhat longer measurement period.

To conclude, this work indicates the CBGS model can accurately estimate the storage and recirculation of gases using only an arterial input of blood flow and gas concentration. Coupling this model to models of lung physiology should allow a more integrated approach to cardiopulmonary gas transport, and this offers opportunities to improve physiological assessment of cardiopulmonary function.

## APPENDIX

This appendix lists the details of the circulatory branches used to calculate each compartment’s arterial volume in Table A1. The vessel identification number provides its location in the condensed circulatory model illustrated in Fig. 2. The data are based on the model of Ref. 14.

**Table A1.** Standard man arterial tree

Vessel No.	Artery Name	Length /cm	Radius /cm	Volume /mL
*Myocardium*				
0	Right coronary	5.3	0.16	0.40
0	Left main coronary	0.5	0.24	0.09
0	Left anterior descending coronary	4.7	0.13	0.26
0	Left circumflex	2.6	0.17	0.22
*Great vessels*				
1	Ascending aorta	4	1.45	26.42
1	Aortic arch (1)	2	1.12	7.88
2	Aortic arch (2)	3.9	1.07	14.03
2	Thoracic aorta (1)	5.2	1.00	16.34
3	Thoracic aorta (2)	10.4	0.95	29.49
4	Abdominal aorta (1)	5.3	0.87	12.60
5	Abdominal aorta (2)		0.57	10.82
6	Left subclavian artery	3.4	0.42	1.88
7	Brachiocephalic artery	3.4	0.62	4.11
*Left lower body and legs*				
8	Common iliac	5.8	0.52	4.93
8	External iliac (1)	8.3	0.29	2.19
8	Internal iliac	5	0.20	0.63
8	Abdominal aorta (2)	10.6	0.27	1.40
8	Femoral artery (1)	12.7	0.24	2.30
8	Profundis artery	12.6	0.23	2.09
8	Femoral artery (2)	12.7	0.24	2.30
8	Popliteal artery	18.8	0.20	2.36
8	Anterior tibial artery (1)	2.5	0.13	0.13
8	Anterior tibial artery (2)	30	0.10	0.94
8	Posterior tibial artery	32.2	0.18	3.28
8	Peroneal artery	31.8	0.13	1.69
*Right lower body and legs*				
8	Vessels as for left lower body and legs			
*Left upper body and arm*				
9	Subclavian artery	6.8	0.40	3.42
9	Internal mammary	15	0.10	0.47
9	Costo-cervical artery	5	0.10	0.16
9	Axilliary artery (1)	6.1	0.36	2.48
9	Suprascapular	10	0.20	1.26
9	Thyrocervical	5	0.10	0.16
9	Thoraco-acromial	3	0.15	0.21
9	Axillary artery (2)	5.6	0.31	1.69
9	Circumflex scapular	5	0.10	0.16
9	Subscapular	8	0.15	0.57
9	Brachial artery (1)	6.3	0.28	1.55
9	Profunda brachi	15	0.15	1.06
9	Brachial artery (2)	6.3	0.26	1.34
9	Brachial artery (3)	6.3	0.25	1.24
9	Superior ulnar collateral	5	0.07	0.08
9	Inferior ulnar collateral	5	0.06	0.06
9	Brachial artery (4)	4.6	0.24	0.83
9	Ulnar artery (1)	6.7	0.21	0.93
9	Radial artery	11.7	0.16	0.94
9	Interossea artery	7.9	0.09	0.20
9	Radial artery	11.7	0.16	0.94
9	Ulnar artery (2)	17	0.19	1.93
*Right upper body and arm*				
10	Vessels as for left upper body and arm			
*Left head*				
24	Superior thyroid artery	4	0.07	0.06
24	External carotid	5.9	0.18	0.60
24	Lingual artery	3	0.10	0.09
24	Facial artery	4	0.10	0.13
24	External carotid (1)	5.9	0.13	0.31
24	External carotid (2)	5.9	0.08	0.12
24	Superficial temporal	4	0.06	0.05
24	Maxilliary artery	5	0.07	0.08
*Right head*				
25	Vessels as for left head			
*Kidneys*				
11	Left renal artery	3.2	0.26	0.68
11	Right renal artery	3.2	0.26	0.68
*Liver*				
12	Coeliac artery	1	0.39	0.48
13	Hepatic artery	6.6	0.22	1.00
*Gastrointestinal tract and spleen*				
14	Gastric artery	7.1	0.18	0.72
14	Splenic artery	6.3	0.28	1.55
15	Superior mesenteric	5.9	0.43	3.43
16	Inferior mesenteric	5	0.16	0.40
17	Portal vein	5.8	0.50	4.56
*Left brain*				
18	Left common carotid	20.9	0.37	8.99
22	Internal carotid	11.8	0.15	0.83
22	Middle cerebral	3	0.06	0.03
22	Cerebral artery	5.9	0.08	0.12
22	Opthalmic artery	3	0.07	0.05
20	Vertebral artery	14.8	0.19	1.68
*Right brain*				
19	Right common carotid	17.8	0.37	7.66
23	Internal carotid	11.8	0.15	0.83
23	Opthalmic artery	3	0.07	0.05
23	Cerebral artery	5.9	0.08	0.12
23	Middle cerebral	3	0.06	0.03
21	Vertebral artery	14.8	0.19	1.68

## GRANTS

This work was supported by the National Institute for Health Research (NIHR) Oxford Biomedical Research Center (BRC). The views expressed are those of the authors and not necessarily those of the NHS, the NIHR, or the Department of Health. S.M.-E. was supported by a British Heart Foundation Doctoral Training Center studentship at the University of Oxford.

## DISCLOSURES

No conflicts of interest, financial or otherwise, are declared by the authors.

## AUTHOR CONTRIBUTIONS

P.A.R. conceived research; S.M.-E. and P.A.R. designed the model; S.M.-E. and C.J.F. wrote the software; S.M.-E. and G.R. conducted validation experiments; S.M.-E., G.A.D.R., and P.A.R. reviewed results; S.M.-E. and P.A.R. drafted manuscript; all authors edited, reviewed, and approved final version of manuscript.
